# Interplay in the Selection of Fluoroquinolone Resistance and Bacterial Fitness

**DOI:** 10.1371/journal.ppat.1000541

**Published:** 2009-08-07

**Authors:** Linda L. Marcusson, Niels Frimodt-Møller, Diarmaid Hughes

**Affiliations:** 1 Microbiology Programme, Department of Cell and Molecular Biology, Biomedical Center, Uppsala University, Uppsala, Sweden; 2 National Center for Antimicrobials and Infection Control, Statens Serum Institut, Copenhagen, Denmark; Emory University, United States of America

## Abstract

Fluoroquinolones are antibacterial drugs that inhibit DNA Gyrase and Topoisomerase IV. These essential enzymes facilitate chromosome replication and RNA transcription by regulating chromosome supercoiling. High-level resistance to fluoroquinolones in *E. coli* requires the accumulation of multiple mutations, including those that alter target genes and genes regulating drug efflux. Previous studies have shown some drug-resistance mutations reduce bacterial fitness, leading to the selection of fitness-compensatory mutations. The impact of fluoroquinolone-resistance on bacterial fitness was analyzed in constructed isogenic strains carrying up to 5 resistance mutations. Some mutations significantly decreased bacterial fitness both *in vitro* and *in vivo*. We identified low-fitness triple-mutants where the acquisition of a fourth resistance mutation significantly increased fitness *in vitro* and *in vivo* while at the same time dramatically decreasing drug susceptibility. The largest effect occurred with the addition of a *parC* mutation (Topoisomerase IV) to a low-fitness strain carrying resistance mutations in *gyrA* (DNA Gyrase) and *marR* (drug efflux regulation). Increased fitness was accompanied by a significant change in the level of *gyrA* promoter activity as measured in an assay of DNA supercoiling. In selection and competition experiments made in the absence of drug, *parC* mutants that improved fitness and reduced susceptibility were selected. These data suggest that natural selection for improved growth in bacteria with low-level resistance to fluoroquinolones could in some cases select for further reductions in drug susceptibility. Thus, increased resistance to fluoroquinolones could be selected even in the absence of further exposure to the drug.

## Author Summary

The increasing frequency of human pathogens resistant to important classes of antibiotics poses a serious and growing challenge for medicine and society. We need improved strategies to reduce the rate of resistance development, for established and novel drugs, based on knowledge of the factors that drive the increase in resistance. Resistance to fluoroquinolones in most bacteria develops via a series of sequential genetic changes affecting several different genes. These are selected and enriched in bacterial populations by exposure to the drug. Relevant factors driving this increase include overuse, and inappropriate use, of these drugs. In this paper we show that mutant bacteria with low-level resistance (not itself a problem to treat with standard drug doses) can evolve by natural selection (for improved growth rate) to acquire mutations that dramatically increase their level of drug resistance. This means that we may need to consider how to reduce inappropriate drug use that can enrich for bacteria with low-levels of resistance, because at that stage some of the mutant bacteria in the population may continue to evolve higher level resistance even in the absence of any further drug exposure.

## Introduction

Fluoroquinolones are potent antibacterial drugs [1] that bind to bacterial type II topoisomerases (DNA gyrase and topoisomerase IV) when they are in complex with DNA. The drugs inhibit chromosome re-ligation after enzyme-mediated cleavage [2]. Fluoroquinolones are effective against many bacteria including invasive *E. coli*, but resistance is increasing with 28 of 29 countries in Europe reporting a significant rise between 2001–2007 [3]. The rapid increase is surprising because clinically relevant levels of resistance in *E. coli* require multiple genetic changes, including mutations altering topoisomerases and up-regulating drug efflux [4], changes that are associated with reduced bacterial fitness *in vitro* and *in vivo* [5]–[7]. Inappropriate use of fluoroquinolones, or co-selection of resistant bacteria with the use of other antimicrobial drugs [8] may be factors driving the increase in resistance but these may not be the sole causes [9]. For example, resistant isolates of *E. coli* increased from ∼7–19% between 2001 and 2007 in the UK [3], while outpatient fluoroquinolone use remained unchanged from 1997–2003 [9]. To develop an effective strategy to restrict the increase in resistance frequency will require that we have a full understanding of the factors driving the increase. The aim of this paper was to investigate whether selection for improved fitness in bacteria might itself be a factor promoting increased resistance.

The fitness costs of drug resistance can be reduced by selection of low-cost mutations or by the accumulation of secondary fitness-compensating mutations that do not reduce resistance. During experimental evolution of clinical isolates of *E. coli* for decreased susceptibility to fluoroquinolones most lineages (16/18) suffered reduced growth competitiveness after only two or three selection steps [5]. However, lineages selected for further decreases in susceptibility were occasionally associated with a relative restoration of fitness [5]. A similar reversal was noted in some constructed strains of *Streptococcus pneumoniae* carrying one or two resistance mutations [10]. These data suggested that some resistance mutations might be selected because they decrease susceptibility to the drug and simultaneously reduce the fitness costs associated with existing resistance mutations. No cause for the phenomenon has been demonstrated, and it's relevance to bacterial fitness *in vivo* in not clear. To examine the phenomenon we constructed isogenic strains carrying various combinations of five resistance mutations found commonly in fluoroquinolone-resistant clinical *E. coli*, and measured their drug-susceptibility and fitness. The relationships between the number of resistance mutations and bacterial fitness were complex and the addition of a resistance mutation was shown in some cases to improve bacterial fitness. These findings have implications for the evolution of fluoroquinolone resistance in the absence of antibiotic exposure.

## Results

### Resistance and fitness in clinical isolates: the paradox

The *E. coli* urinary tract infection isolate C1186 [4] is highly-resistant to fluoroquinolones (MIC for ciprofloxacin ≥32 µg/ml) and carries resistance mutations altering topoisomerases (*gyrA* Ser83→Leu, Asp87→Asn; *parC* Ser80→Ile), and up-regulating drug efflux (*marOR* small deletion, and amino acid substitution; *acrR* IS1 insertion). These 5 mutations are typical of highly resistant clinical isolates [4]. C1186 has a growth rate similar to a laboratory wild-type. Thus, these resistance mutations may individually be low-cost, as found for some rifampicin-resistant patient isolates [11],[12] or the strain may carry additional fitness-compensatory mutations [7]. A third possibility is that some resistance mutations reduce existing fitness costs while simultaneously decreasing susceptibility to the drug. This last possibility is highly relevant to the multi-step nature of fluoroquinolone-resistance development. We constructed 28 isogenic derivatives of the wild-type MG1655, each mimicking in part the complex resistance genotype of C1186 (Table 1). Mutations were initially isolated spontaneously (*gyrA*, *parC*) or constructed by λ-red recombineering (Δ*marR*, Δ*acrR*) and then separately introduced into MG1655 by P1 transduction. The *gyrA* and *parC* mutations were transduced by selection for a linked genetic marker (introduced using λ-red recombineering, see Materials and Methods) and at least 20 transductants of each cross were subsequently screened by phenotype (MIC) and DNA sequencing for the linked mutation. In every case only two phenotypic and genotypic classes were found, showing that the *gyrA* and *parC* mutations were not associated with other mutations.

**Table 1:** Genotypes, susceptibility, and fitness of isogenic strains.

Strain	*gyrA*1	*gyrA*2	*parC*	*marR*	*acrR*	MIC^b^	Fitness (SD)^c^	P^d^	N^e^
MG1655	-	-	-	-	-	0.016	1.00 (0.01)	-	5
LM378	S83L	-	-	-	-	0.38	1.01 (0.03)	0.37	6
LM534	-	D87N	-	-	-	0.25	0.99 (0.03)	0.57	7
LM792	-	-	S80I	-	-	0.016	0.99 (0.01)	0.59	5
LM202	-	-	-	Δ	-	0.032	0.83 (0.03)	<0.0001	5
LM351	-	-	-	-	Δ	0.047	0.91 (0.02)	<0.0001	5
LM625	S83L	D87N	-	-	-	0.38	0.97 (0.03)	0.04	8
LM862	S83L	-	S80I	-	-	1	0.98 (0.03)	0.18	6
LM421	S83L	-	-	Δ	-	1	0.86 (0.03)	<0.0001	5
LM647	S83L	-	-	-	Δ	0.5	0.95 (0.04)	0.02	11
LM1124	-	D87N	S80I	-	-	0.38	1.02 (0.02)	0.04	8
LM538	-	D87N	-	Δ	-	1	0.83 (0.03)	<0.0001	5
LM592	-	D87N	-	-	Δ	0.38	0.92 (0.03)	0.0001	10
LM367	-	-	-	Δ	Δ	0.125	0.82 (0.04)	<0.0001	5
LM693	S83L	D87N	S80I	-	-	32	1.01 (0.02)	0.48	13
LM695	S83L	D87N	-	Δ	-	0.75	0.79 (0.03)	<0.0001	10
LM691	S83L	D87N	-	-	Δ	0.75	0.93 (0.03)	0.0002	13
LM871	S83L	-	S80I	Δ	-	6	0,86 (0.03)	<0.0001	10
LM873	S83L	-	S80I	-	Δ	3	0.92 (0.04)	0.0005	10
LM882	-	D87N	S80I	Δ	-	0.75	0.84 (0.05)	<0.0001	14
LM1125	-	D87N	S80I	-	Δ	0.5	0.95 (0.01)	<0.0001	8
LM709	S83L	-	-	Δ	Δ	1.5	0.78 (0.04)	<0.0001	5
LM595	-	D87N	-	Δ	Δ	1.5	0.60 (0.09)	<0.0001	10
LM701	S83L	D87N	-	Δ	Δ	2	0.66 (0.07)	<0.0001	10
LM707	S83L	D87N	S80I	Δ	-	32	0.89 (0.03)	<0.0001	10
LM703	S83L	D87N	S80I	-	Δ	32	0.94 (0.03)	0.0009	14
LM875	S83L	-	S80I	Δ	Δ	8	0.71 (0.04)	<0.0001	5
LM878	-	D87N	S80I	Δ	Δ	2	0.72 (0.05)	<0.0001	10
LM705	S83L	D87N	S80I	Δ	Δ	32	0.68 (0.07)	<0.0001	5

a Genotype. Strains are isogenic to MG1655 and carry only the mutations shown.

b MIC for ciprofloxacin (µg/ml).

c Fitness. Mean fitness per generation, relative to wild-type, SD, standard deviation (within parentheses) measured in pair wise competition against the isogenic wild-type (Δ*araBC*).

d P. Statistical significance of difference in fitness relative to the wild-type (Students t-test (95% confidence limit, two-tailed P-value).

e N. Number of independent competition experiments on which the fitness value is based.

### Susceptibility of isogenic mutant strains

**Figure 1:**
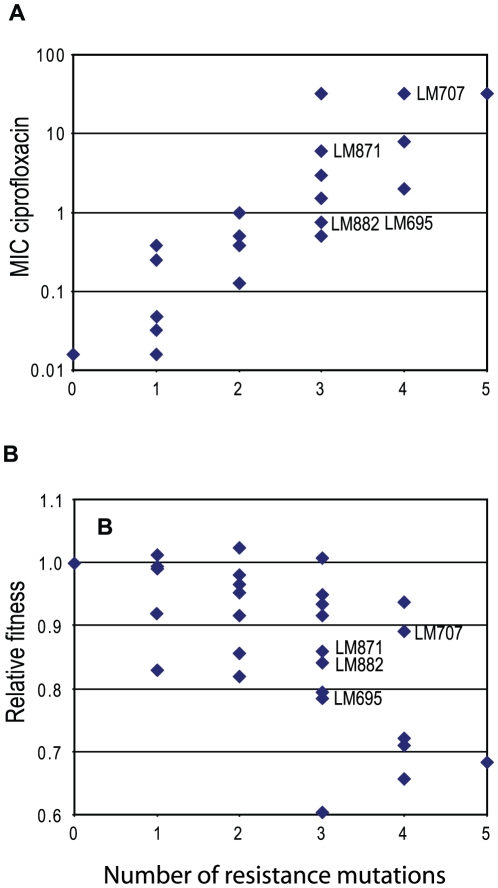
Resistance and fitness of constructed strains. For the wild-type and each of the 28 constructed isogenic strains the MIC for ciprofloxacin (part A) and the relative fitness per generation in *in vitro* growth competition (part B) is shown as a function of the number of resistance mutations per strain. Each diamond symbol represents one strain. In some cases more than one symbol occupies the same space. Strain identification numbers are shown for four strains of particular interest (Table 2).

MIC for ciprofloxacin was measured for each strain (Table 1). The margin of error of MIC values is ±1 half-doubling step. Accordingly, any change that is at least 2-fold is significant. Single mutations in *gyrA*, S83L and D87N, increased MIC 24-fold and 16-fold respectively. Knockout mutations in *marR* and *acrR* increased MIC only 2–3-fold, while the substitution S80I in *parC* had no effect on MIC. Double mutation combinations had MIC's that were 8–64-fold wild-type level, with the combination Δ*marR*+Δ*acrR* having the smallest increase. Certain combinations with *parC* were not tested because in *E. coli parC* mutations only selected after the prior occurrence of a mutation in *gyrA*. Triple mutation combinations have MIC's that were 31–2000-fold wild-type level. Most strains with three resistance mutations (5/9), and all strains with 4 or 5 mutations had MIC's above the 1 µg/ml breakpoint that defines clinical resistance in Europe [13] equivalent to 64-fold wild-type MIC in these strains. On average there was a positive correlation between the number of resistance mutations carried by a strain and the MIC for ciprofloxacin (Figure 1).

### Fitness of isogenic resistant strains

The 28 mutant strains were tested in growth competitions against wild-type to measure their Malthusian fitness [14],[15] as a function of the resistance mutations they carried (Table 1). The fitness value associated with having either one or two resistance mutations ranged from ∼1 down to 0.82 per generation. Some single mutations (*gyrA* S83L, *gyrA* D87N, and *parC* S80I) were statistically neutral whereas others (Δ*marR* and Δ*acrR*) caused a significant reduction in fitness (0.83 and 0.91 per generation, respectively). Strains carrying three resistance mutations had fitness values that ranged from ∼1 down to as low as 0.60, with 8/9 strains suffering a fitness deficit of ≥5% per generation. Interestingly, the addition of a fourth resistance mutation to these strains restricted the minimum fitness value to 0.66 per generation, higher than that measured with three mutations (Figure 1). When all five resistance mutations were present the fitness value was 0.68. Thus, some strains carrying 4 or 5 resistance mutations have a higher fitness than some strains carrying only 3 mutations. The major negative effect on fitness was associated with the presence of the *marR* and *acrR* mutations. Thus, fitness did not decrease as a simple function of the number of resistance mutations, but instead depended critically on the nature of those mutations.

### For some strains an increased MIC correlates with improved fitness

In general the addition of a resistance mutation to a strain was either neutral with respect to MIC and fitness, or it caused an increased MIC and / or decreased fitness (Figure 1, Table 1). Across all the strains constructed decreased fitness was very strongly associated with the presence of one or both efflux mutations. In contrast, strain LM693 (*gyrA* S83L, D87N; *parC* S80I) has high level resistance (MIC 32) with no significant reduction in fitness relative to the wild-type (Table 1). LM693 shows that it is possible to evolve high level resistance with no, or minimal, fitness costs. However, mutations up-regulating drug efflux are highly relevant to resistance evolution because they arise at a rate hundreds of times higher than mutations in the structural genes for topoisomerases. This is because the genetic target for knockout mutations in efflux regulating genes is much larger than the target for the specific amino acid substitutions required in topoisomerase genes. Thus, even though, as shown here, efflux mutations are fitness-costly and contribute relatively small increases in resistance, they occur very frequently, and are found in many resistant clinical isolates [4]. For three low-fitness strains, each carrying three resistance mutations including a *marR* mutation, the addition of an extra resistance mutation increased both MIC and fitness: LM695→LM707; LM882→LM707; and LM871→LM707 (Figure 1 and Table 1). In these strains the added mutation affected *gyrA* or *parC*. The increased fitness was statistically significant (Students t-test, two-tailed, p<0.05) in two of the three cases, LM695>LM707 and LM882→LM707 (Table 2). The robustness of these results was verified by independently reconstructing each of these strains and re-measuring their MIC and fitness values. No significant differences were found from the original values. In addition, we tested these critical strains by de-construction experiments: replacing *gyrA* and/or *parC* mutations with equivalent wild-type genes and determining that the MIC and fitness values of the de-constructed strains were as expected. Based on these two experiments, re-constuction, and de-construction, we are confident that the isogenic strains do not carry any additional mutations affecting MIC or fitness. Thus, the addition of a single resistance mutation can increase growth fitness by 5–10% per generation and simultaneously increase MIC more than 40-fold (Table 2).

**Table 2:** Single mutations decrease drug susceptibility and increase fitness.

Strain	*gyrA*1	*gyrA*2	*parC*	*marR*	MIC^b^	Fitness (SD)^c^	P^d^	N^e^
LM695	S83L	D87N	-	Δ	0.75	0.79 (0.03)	<0.0001	10
LM707	S83L	D87N	S80I	Δ	32	0.89 (0.03)		10
LM882	-	D87N	S80I	Δ	0.75	0.84 (0.05)	0.014	14
LM707	S83L	D87N	S80I	Δ	32	0.89 (0.03)		10
LM871	S83L	-	S80I	Δ	6	0.86 (0.03)	0.05	10
LM707	S83L	D87N	S80I	Δ	32	0.89 (0.03)		10

a Genotype; All strains are isogenic to MG1655 and carry only the mutations shown.

b MIC for ciprofloxacin (µg/ml).

c Fitness; Mean fitness per generation (relative to wild-type). SD, standard deviation (within parentheses) measured in pair wise competition against the isogenic wild-type.

d P; Significance of fitness difference between the strains in each pair, (Students t-test, two-tailed P-value).

e N; Number of independent competition experiments on which the fitness value is based.

### Fitness in a mouse infection model

**Figure 2:**
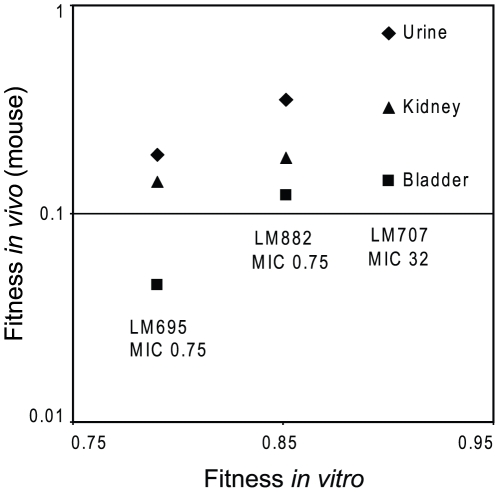
Fitness *in vitro* as a function of fitness *in vivo* in a mouse UTI model. Samples from urine, kidney, and bladder are illustrated with black diamonds, triangles, and squares, respectively. The addition of one extra resistance mutation to either LM695, or LM882 created LM707, with increased fitness *in vitro* and *in vivo* and an increase in MIC for ciprofloxacin from 0.75 to 32 µg/ml.

The data in the previous section showed that the addition of a fourth resistance mutation to either LM695 or LM882 could significantly increase competitive growth fitness measured *in vitro*. This result would be more interesting from a clinical viewpoint if the measured increase in fitness was not exclusively an *in vitro* phenomenon. To test this competition experiments were made in a mouse UTI infection model [16]. Each of the strains (LM695, LM883, and LM707) was competed against the isogenic wild-type in the mouse model and relative fitness expressed as a competitive index (C.I.).Three different measures of C.I. could be obtained in this model: from the urine; the bladder; and the kidneys. For each strain and tissue there was a clear positive correlation between relative fitness *in vivo* and *in vitro* (Figure 2). Thus, the relative order of fitness values of these strains, initially measured *in vitro*, was confirmed in the physiologically more complex *in vivo* environments.

### Fitness compensation and changes in DNA supercoiling

The *marR* mutation makes the single largest contribution to loss of fitness in these 28 strains (Table 1). The *marR* mutation alone reduced fitness to 0.83±0.3 (LM202), and the average fitness of all strains carrying *marR* is 0.76 (SD 0.08). MarR protein regulates, directly and indirectly, transcription of many genes [17] and the reduction in fitness associated with loss of function mutations in *mar* is most likely because of disruption of gene regulation. This suggests a possible mechanism of fitness compensation associated with resistance mutations in topoisomerase genes. Thus, some topoisomerase mutations that alter chromosomal supercoiling levels may partially restore expression of growth-limiting gene(s) regulated by *mar*, reversing the fitness deficit. This hypothesis predicts that the improvement in fitness measured in LM695→LM707 should be associated with a change in global supercoiling level. This was tested by electrophoresis of pUC18 and pUC19 plasmids purified from MG1655, LM695 and LM707 in chloroquine-agarose gels [18]. With this method we were unable to detect differences in plasmid topoisomer patterns (data not shown). We also tested for differences in supercoiling using a reporter gene assay [19],[20]. This was done by introducing plasmids carrying a luciferase reporter gene fused to each of two different promoters (p*topA* and p*gyrA*) whose expression is sensitive to, respectively, increased or decreased supercoiling levels [20]. In this assay the ratio (p*topA* / p*gyrA*) of reporter gene expression from these two promoters, defined as the quotient of supercoiling (Qsc), is a measure of the relative level of negative supercoiling in isogenic strains [19],[20]. Qsc was 2.9 (SD 0.7) for MG1655 (n = 6), 2.6 (SD 0.7) for LM695 (n = 12), and 3.6 (SD 1) for LM707 (n = 13). In this assay neither of the mutant strains differed significantly from the wild-type, but LM695 and LM707 differed significantly from each other (P value 0.01, Students t-test, two tailed). From the expression data it was clear that the major cause of this difference in Qsc between LM695 and LM707 was due to a 25% decrease in expression from the *gyrA* promoter construct in LM707 relative to LM695. Thus the introduction of the *parC* S80I mutation to make strain LM707 caused a significant increase in the Qsc, coincident with the improvement in fitness. Although we cannot explain the absence of an effect in the chloroquine-agarose assay, the reporter gene assay suggests that some changes in chromosomal supercoiling associated with the acquisition of topoisomerase mutations may provide a mechanism linking bacterial fitness and decreased susceptibility to fluoroquinolones. However, an alternative explanation is that the combination of mutations in LM707 reduces expression of *gyrA* by a mechanism that does not change global supercoiling levels, and that it is a consequence of the reduced expression of *gyrA* that causes the increase in growth fitness. Additional experiments will be required to distinguish between these models.

### Selection of improved-fitness highly-resistant mutants

In the sections above it was shown that the transfer of the *parC* S80I mutation into LM695 increased its MIC for ciprofloxacin and its competitive growth fitness versus the wild-type. This predicted that the resulting constructed strain, LM707, should outcompete LM695 in a head-to-head competition, and also raised the following questions: (i) could a mutant with an increased MIC for ciprofloxacin and a higher growth fitness be selected spontaneously from LM695 in the absence of drug; and (ii) would such a mutant be exclusively associated with of the acquisition of the *parC* S80I mutation. These predictions and questions were addressed experimentally. First, in head-to-head growth competition experiments in LB medium with no drug, LM707 (four resistance mutations) outcompeted LM695 (three resistance mutations), gaining ∼8% per generation, in good agreement with the relative differences in growth fitness of each strain versus the wild-type (Table 1). Second, 96 independent lineages of LM695 were grown in rich medium in the absence of drug for 4 growth cycles. Each growth cycle was inoculated with 2×10^6^ cfu and grown to a total of 2×10^8^ cfu (∼7 generations per growth cycle) in a volume of 200 µL. An aliquot of 5×10^6^ cfu from each lineage was tested, after the completion of 2, 3, and 4 cycles of growth, for the presence of resistant mutants on solid medium (3 µg/mL ciprofloxacin, 4×MIC). Growth of LM695 is completely inhibited on 3 µg/mL ciprofloxacin and the spontaneous mutation rate to resistance on this media, measured in fluctuation tests, is 2×10^−8^. Accordingly, we expected that virtually none of the 96 independent lineages would contain a resistant mutant at the initiation of the experiment, but that a small number of resistant mutants would arise in each lineage during each growth cycle. If these mutants out-competed the parental LM695 they would be expected to increase relative to LM695, and thus have a greater probability of being transferred to the next growth cycle. The number of lineages from which resistant mutants were obtained was found to increase with successive growth cycles: from 2/96 (cycle 2)→8/96 (cycle 3)→15/96 (cycle 4). Because the spontaneous mutation rate to resistance (2×10^−8^) if much lower than the number of cells being tested from each lineage (5×10^6^) it is very unlikely that these mutants arose on the selective media. Instead, the most reasonable conclusion is that the resistant mutants arose spontaneously and randomly during the growth of lineages in the absence of drug and were enriched because they out-competed the parent population. Three random drug-resistant mutants were chosen from independent lineages and tested by DNA sequencing for the presence of mutations in *gyrA* and *B*, in *parC* and *E*, and in *marR* and *acrR*. In each case the mutations originally present in LM695 were confirmed and in addition each of the mutants was found to have acquired a new mutation in *parC* (S80R in two cases, and E84K in one case). The MIC CIP of each of the mutants had increased from 0.75 to >32 µg/mL, and the exponential doubling time in rich medium had increased significantly, by ∼10% per generation (Table 3). Thus, the selection of a spontaneous *parC* mutation in LM695 decreased its susceptibility to ciprofloxacin and increased its growth rate. From these experiments we concluded that the phenotypes generated directly by strain construction (LM695→LM707; reduced drug susceptibility and increased growth fitness) could also be generated by a variety of spontaneous mutations, and that the growth advantage phenotype could be enriched by growth selection in the absence of drug.

**Table 3:** Selection of LM695 to faster growth and increased MIC.

	Dt^a^	P value^b^	Mutation^c^	MIC^d^
LM695	23.0±0.7	-	-	0.75
LM1179	20.8±1.0	<0.0001	*parC* S80R	>32
LM1198	20.8±0.5	<0.0001	*parC* S80R	>32
LM1199	21.2±0.7	<0.0001	*parC* E84K	>32

a Dt; doubling time in min in LB medium at 37°C.

b P-value; Significance of the difference in generation time relative to LM695, the parental strain, Students t-test for independent samples, n = 10 for each strain.

c Mutation; Identity of the new mutation identified in each evolved strain.

d MIC; for ciprofloxacin (µg/ml) of LM695 and each of the evolved strains.

## Discussion

A set of 28 isogenic *E. coli* strains was constructed and used to measure the relationship between the accumulation of fluoroquinolone resistance mutations, drug susceptibility, and growth fitness. The question was whether the accumulation of up to five resistance mutations, commonly found in resistant clinical isolates, would progressively reduce bacterial fitness. Most of the published data on the relationship between drug resistance and bacterial fitness would predict two possibilities: (i) that these mutations would cause little or no fitness cost, explaining their high frequency among resistant isolates; or (ii) that their accumulation would cause a progressive decrease in bacterial fitness, and require additional fitness-compensating mutations to restore fitness [6],[7]. The data from this study support, in part, each of these scenarios (Table 1, and Figure 1). However, they also revealed, for some combinations of resistance mutations, a positive relationship between reduced drug-susceptibility and increased bacterial fitness. This positive relationship could be another driving force in the development of increased resistance to these antibacterial drugs. Although this may be the first demonstration in bacteria that in the absence of an antimicrobial, selection can increase resistance to that antimicrobial, a similar phenomenon has been reported for HIV resistance to a protease inhibitor [21]. Thus, the phenomenon described here may have a broad biological significance.

**Figure 3:**
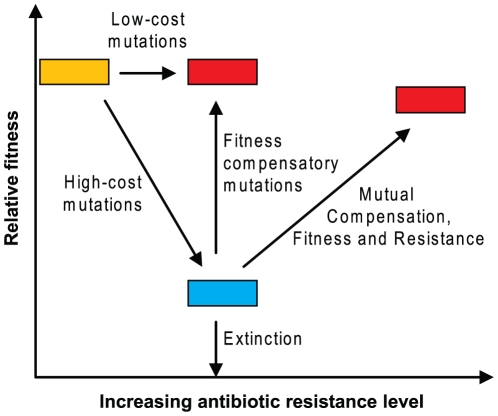
A general model illustrating evolutionary paths for a lineage under antibiotic selection. Mutual compensation, the acquisition of a mutation that simultaneously improves fitness and reduces susceptibility to the antibiotic, is the pathway presented in this manuscript.

The main conclusions from the data set were the following:

1. Some resistance mutations, individually or when incorporated in an already mutant strain, reduced drug susceptibility without significantly reducing fitness. Other mutations were individually or in combination associated with significant fitness costs. This suggests that selection for low-cost mutations, and/or compensating mutations could be selective forces determining the frequency of particular mutations individually or in combination. The *marR* mutation had the largest negative effect on fitness.
2. In some cases, the addition of a resistance mutation to an already low-fitness mutant strain caused a further reduction in drug susceptibility **and** an increase in relative fitness (Figure 3). The improved fitness of these strains was also measured *in vivo*. Thus, particular combinations of resistance mutations could potentially be selected either because they reduce drug susceptibility, and/or because they improve the relative fitness of the mutant strain.
3. The positive effect of these combinations of resistance mutations on fitness was accompanied by a significant change in the level of *gyrA* expression measured in a reporter gene assay of supercoiling. This suggests that reversing the negative effects of the *marR* mutation on gene regulation may be a mechanism for expression of the phenotype.
4. Growth of a low-fitness strain in the absence of drug selected for mutants with increased growth fitness and reduced drug susceptibility. Because each culture was initiated with a pure clone, these mutants must have arisen spontaneously during the growth of the cultures and been selected because they had a competitive advantage. This showed that the phenotype could be selected in the absence of drug, and that it was not limited to a unique combination of mutations.

Molecular details of how particular *parC* and *gyrA* mutations together improve the fitness of a resistant strain with a *marR* mutation are beyond the scope of this study but we can suggest the outlines of a model. Growth rate depends on the rate of transcription regulated in accordance with physiological demands [22]. The MarR protein regulates, directly and indirectly, the transcription of many genes [17] and it is probable that the severe reduction in fitness associated with Δ*marR* (Table 1) is because it causes inappropriate patterns of transcription regulation. We suggest that specific mutant forms of DNA gyrase and topoisomerase IV, possibly by acting in concert to influence the level of superhelicity in DNA, restore appropriate levels of gene expression at some loci where the loss of MarR regulation has a negative impact on growth rate [23],[24].

The particular *gyrA* and *parC* resistance mutations studied here are clinically relevant, being among the most common found in fluoroquinolone-resistant clinical isolates of *E. coli* [4]. Among 30 resistant UTI isolates analyzed: 30/30 had the *gyrA* S83L mutation; 18/30 had the double mutation *gyrA* S83L, D87N; 22/30 had the *parC* mutation S80I; and 15/30 had the triple combination *gyrA* S83L, D87N, *parC* S80I (23/30 had some form of triple mutation combination including other substitutions at position 87 of *gyrA* and/or position 80 of *parC*). Thus, the combination of target mutations studied here is typical of resistant clinical isolates. As measured by organic solvent tolerance, drug efflux was phenotypically up-regulated in 15/30 of these resistant isolates, associated in most cases with mutations in *acrR* and/or *marOR* [4]. The observed frequency of the efflux phenotype is very low relative to the frequency of specific gyrase and topoisomerase mutations in the same isolates, and given the much higher expected probability of mutations that knock out the function of efflux regulator genes it suggests selection against such mutations. This under-representation is consistent with our finding that mutations up-regulating efflux pumps carry significant fitness costs. The growth fitness of clinical isolates cannot be meaningfully compared with the data presented in this paper, in part because clinical isolates are not isogenic, and in part because clinical isolates will already have evolved to ameliorate the fitness costs, if any, associated with their resistance determinants. We believe that our data provide an insight into the likely initial effects on relative fitness and drug susceptibility of different pathways of resistance development. In particular, that one consequence of a following a high-probability but low-fitness evolutionary pathway may be that one or more steps may be driven by selection for increased fitness in the absence of drug exposure.

How fluoroquinolone resistance evolves in nature will depend on the genotype being selected and on the selective environment [25] but it is likely include mutational steps that reduce bacterial fitness. Bacteria that progress down an evolutionary path with reduced fitness relative to a competing population may be driven to extinction, or may, given the opportunity by mutation, acquire a change that increases their relative fitness thus improving their chances of survival. Evolutionary paths that could be taken on the road to extinction or antibiotic resistance are outlined in Figure 3. The low-fitness mutants LM695 and LM882 each have MICs that lie under the resistance breakpoint for ciprofloxacin [26],[27]. Such mutants are at a critical stage in resistance development: having low fitness they may be driven to extinction by natural selection; being under the resistance breakpoint they may avoid detection in a clinical setting; however, they are, as shown here, only one mutational step away from a high-level resistance phenotype with increased fitness, without additional exposure to the drug. The magnitude of these co-selected changes in fitness and susceptibility are significant (Table 2). These data argue in favor of testing anti-mutant dosing strategies, or other measures that could prevent the enrichment of low-level resistant mutants [28].

## Materials and Methods

### Strains and growth conditions

C1186 is a multiply mutant fluoroquinolone resistant UTI isolate previously described [4]. *E. coli* K12 MG1655 wild-type was the starting strain for all constructions (Table 1). Liquid growth medium was Luria broth (LB) while solid medium was Luria-Bertani agar (LA). Strains were grown at 37°C. Ciprofloxacin (Bayer HealthCare AG, Wuppertal, Germany) was dissolved in 0.1 M NaOH at 100 µg/mL then further diluted in LA in selective plates.

### MIC determinations

MIC was determined by Etest (AB BIODISK, Solna, Sweden) on Mueller-Hinton agar plates incubated for 16 to 18 h at 37°C with quality control reference strains [4] as recommended by the Clinical and Laboratory Standards Institute (www.clsi.org).

### Selection of mutations and strain construction

Spontaneous resistance mutations in *gyrA* and *parC* were selected sequentially in *E. coli* MG1655 in LB with ciprofloxacin at 2–8-fold MIC and mutations were identified by DNA sequencing. Individual mutations were always moved into a clean genetic background (MG1655) after initial selection. Deletion-replacement mutations in *marR*, *acrR*, *yfaH*, *metC* and *araB* were made by λ-red recombineering [29] in NC397 (a Lac^+^ Nad^+^ derivative of DY329) using PCR amplified linear DNA from pCP16 with an FRT-bounded Tc^R^ cassette [30]. The PCR reaction protocol was 95°C 5 min followed by 30 cycles of 95°C 15 sec, 55°C 20 sec, 72°C 240 sec. PCR primer sequences with details of the deletion-replacement boundaries are shown in Supporting Information (Table S1).

### Construction of isogenic strains

Isogenic derivatives of MG1655 were constructed by phage P1-mediated transduction. Transduction of the deletion-replacement mutations in *marR* and *acrR* was selected directly on LA+Tc. When transducing the *gyrA* and *parC* mutations selection was made for the linked markers, *yfaH*<>Frt::Tc^R^::Frt and *metC*<>Frt::Tc^R^::Frt, each ∼10 kb from *gyrA* and *parC*, respectively. Note that we are using the symbol <> to indicate a replacement generated by λ red homologous recombination technology. After transduction the Tc^R^ marker was removed by Flp-catalyzed excision expressed following transformation with pCP20 [30]. All strain constructs were confirmed by DNA sequencing.

### Growth competition assays

LM347 (Δ*araB*<>FRT) was used as the standard wild type strain in growth competitions against which each of the constructed mutant strains was competed. This strain was tested against its parent MG1655 showing that the Δ*araB* mutation was neutral (relative growth rate 1.002±0.005). To support statistical analysis each competition was tested in at least 5 independent experiments (Table 1). To initiate growth competitions, each strain was grown in LB at 37°C 12 h, mixed in a 1∶1 ratio, diluted 10^−3^ into LB, then grown 23 h to complete a growth cycle. Each successive growth cycle was initiated by diluting the mixture 10^−3^ into LB. Each competition experiment (4 cycles) was made the number of times indicated in Table 1. For some low-fitness strains such as LM595 the mutant population became too low to detect after the second or third cycle of competition. After the initial mixing, and after each growth cycle, appropriate dilutions of the mixture were plated onto MacConkey agar plates containing 1% arabinose. Plates were incubated 37°C overnight. Red (*ara* ^+^) and white (Δ*araB*) colonies were scored. The change in the ratio of mutant/wild-type was used to estimate the selection coefficient per generation of each of the constructed strains according to the formula: *S* = ln2 (mutant/wild-type) / generation [31]. Relative fitness per generation with respect to the wild-type LM347 is defined as *S*+1. Note that the fitness defects could be due to defects at any stage of the growth cycle (lag, exponential, stationary phase).

### The ascending urinary tract infection model in mice

Relative bacterial fitness *in vivo* was measured using an established urinary tract infection model [16]. Details, including mouse strain and ethical permission, are given in Text S1. Competitive index was calculated as the geometric mean of the ratio of mutant/wild-type bacteria isolated from each organ (urine, bladder, kidneys) of 8 mice per experiment, normalised to the ratio at the time of inoculation.

### Growth rate measurements

Exponential doubling times were calculated by measuring the increase in optical density at 600 nm (OD_600_) at 10-min intervals, using a BioscreenC machine (Oy Growth Curves Ab Ltd. Helsinki, Finland).

### Evolution of improved fitness mutants

The mutation rate of LM695 to resistance on LA+3 µg/mL ciprofloxacin (CIP) is 2×10^−8^ measured by fluctuation test. Independent lineages were inoculated in 96×200 µL wells, and grown 24 h at 37°C to ∼10^9^ CFU/mL. 2 µL was transferred to initiate a new growth cycle. 5 µL was plated on LA+3 µg/mL CIP to assay for resistant mutants.

### Supercoiling assay

The relative supercoiling degree was determined using a published assay. The quotient of supercoiling, Qsc [19] is defined as the ratio of luciferase activity from two different supercoiling sensitive promoters, p*topA-luc*, and, p*gyrA-luc*, [20]. Details are given in Text S2.

## Supporting Information

Table S1 Oligonucleotides used to amplify linear PCR fragments for λ-red recombineering
(0.03 MB DOC)

Text S1 The ascending urinary tract infection model in mice.
(0.03 MB DOC)

Text S2 Supercoiling assay.
(0.03 MB DOC)
