# Supplementary material for: Interplay in the Selection of Fluoroquinolone Resistance and Bacterial Fitness
Source: PLoS Pathog. 2009 Aug 7;5(8):e1000541. doi: 10.1371/journal.ppat.1000541 (PMC2714960; doi:10.1371/journal.ppat.1000541)
Supplement: Table S1 — Oligonucleotides used to amplify linear PCR fragments for λ-red recombineering (0.03 MB DOC) [file ppat.1000541.s001.doc]

**Table S1 (Supporting information)**

Oligonucleotides used to amplify linear PCR fragments for λ-red recombineering

**Gene Primer**

*marR*^a^ marF1459

gcgatctgttcaatgaaattattccattgggtcgcttaatccatatggttgaattcgagctcggtacccg

marR1831

cgccgtcaggttttttgttaattcttggtgcaggtcctggccaactaattaagcttcaaaagcgctctga

*acrR*^b^ acrRΔf1

gagatctgggaactgtcagaatccaatattggaattcgagctcggtacccg

acrRΔr1

ttcaatacaatgttttaacgtttgttctatgctatgaccatgattacgcc

*yfaH*^c^ yfaHΔf

aatagtgcgcccagcagataaaatgtgaatttaatcaggcggatacagcggaattcgagctcggtacccg

yfaHΔr

aaagagtaatgttactgcaggatggcgttcatgcgccatcctgtctcatagctatgaccatgattacgcc

*metC*^d^ metCΔf

atattcatgctagtttagacatccagacgtataaaaacaggaatcccgacgaattccagctcggtacccg

metCΔr

taagacttttcacaataaaatgtctgcaaaattgtccaaaagtggcaatggctatgaccatgattacgcc

*araB*^e^ araBΔf

ccacacttcataattatcaaaaatcgtcattatcgtgtccgaattcgagctcggtacccg

araBΔr

actgtttctccatacccgtttttttggatggagtgaaacggctatgaccatgattacgcc

^a^ Nucleotides 11 to 387 (where 1 is the 1^st^ nt of the *marR* start codon GTG) are deleted and replaced with an FRT sequence.

^b^Nucleotides 212 to 450 (where 1 is the 1^st^ nt of the *acrR* start codon ATG) are deleted and replaced with an FRT sequence.

^c^ The complete coding sequence of *yfaH* is deleted and replaced with an FRT sequence.

^d^ The complete coding sequence of *metC* is deleted and replaced with an FRT sequence.

^e^ The complete coding sequence of *araB* is deleted and replaced with an FRT sequence.
