# Supplementary material for: Interplay in the Selection of Fluoroquinolone Resistance and Bacterial Fitness
Source: PLoS Pathog. 2009 Aug 7;5(8):e1000541. doi: 10.1371/journal.ppat.1000541 (PMC2714960; doi:10.1371/journal.ppat.1000541)
Supplement: Text S1 — The ascending urinary tract infection model in mice. (0.03 MB DOC) [file ppat.1000541.s002.doc]

**Text S1**

**The ascending urinary tract infection model in mice.** Female OF-1 mice (Charles River Laboratories) weighing ~30 g were used for all experiments. Mice were housed in cages with 6 mice/cage. Three days before the start and during the study the mice were given 5 % glucose as drinking water, and had otherwise free access to food. Animal experiments were conducted according to approval by the Animal Experiments Inspectorate under the Danish Ministry of Justice (permit 2004/561-835). Bacterial inocula were prepared from fresh overnight colonies grown on 5 % horse blood agar (SSI, Copenhagen) then suspended in saline. The two strains to be competed were mixed in a 1:1 ratio at ~10^9^ CFU/ml then diluted to 1x10^8^ CFU/ml. On the day of experiment and prior to anesthetizing the mice, urine was removed from the bladder by gently pressing the abdomen. Mice were anesthetized by intraperitoneal administration of 0.08 ml of a 5:1.5 mixture of Hypnorm (fentanyl citrate, 0.315 mg/ml; fluanisone, 10 mg/ml) and Stesolid (diazepam, 5 mg/ml). A catheter (1), two mm in diameter, fastened to a syringe containing the bacterial suspension, was inserted via the urethra into the bladder and 50 µl of the inoculum was slowly injected into the bladder. After retracting the catheter the mice were left in the cage. The day after inoculation urine was collected in microfuge tubes and the mice were sacrificed by cervical dislocation. CFU determinations were made immediately on the urine samples by spotting 20 μL aliquots of appropriate dilutions on indicator medium (MacConkey agar with 1% arabinose) to distinguish wild-type from mutant bacteria. The bladder and kidneys were removed aseptically. The bladder was placed in a tube with 0.5 ml saline and the two kidneys in a tube with 1.0 ml saline, and stored at -80°C until homogenization. Each organ sample was homogenized by thawing, adding two steel beads then placing in a TissueLyser for two minutes at 30 Hz. Each homogenized sample was diluted in saline and 20 µL aliquots were applied to MacConkey arabinose indicator agar plates. All plates were incubated for 18 - 24 h at 35°C before colony counting. Competitive index was calculated as the geometric mean of the ratio of mutant/wild-type bacteria isolated from each organ (urine, bladder, kidneys) of 8 mice per experiment, normalised to the ratio at the time of inoculation.

1. Hvidberg H*, et al.* (2000) *Development of a long-term ascending urinary tract infection mouse model for antibiotic treatment studies*. *Antimicrob Agents Chemother* 44:156-163.
