# Supplementary material for: Interplay in the Selection of Fluoroquinolone Resistance and Bacterial Fitness
Source: PLoS Pathog. 2009 Aug 7;5(8):e1000541. doi: 10.1371/journal.ppat.1000541 (PMC2714960; doi:10.1371/journal.ppat.1000541)
Supplement: Text S2 — Supercoiling assay. (0.03 MB DOC) [file ppat.1000541.s003.doc]

**Text S2**

**Supercoiling assay**. The relative supercoiling degree was determined as the quotient of supercoiling, Qsc (1). This is defined as the luciferase activity determined for a strain containing pPHB94, p*topA-luc*, divided by that for the same strain containing pPBH95, p*gyrA-luc*, (2). Compared to an isogenic control strain, higher and lower Qsc values correspond to greater and lesser degrees of negative supercoiling, respectively (1). The plasmids were kindly supplied by Peter Heisig (University of Hamburg, Germany). Bacterial cultures were grown to mid-log phase (OD_600_ ~0.5) then put on ice. 90 μl of culture and 10 μl 1 M K_2_HPO_4_, 20 mM EDTA pH 7.8 were mixed in a microfuge tube and then quick-frozen on dry ice. Cells were thawed to RT in a water bath. 300 μl freshly prepared lysis mix (Luciferase Assay System, Promega Corporation, Madison, WI, USA) was added and cells were incubated at RT for 10 minutes. The lysis mixture was centrifuged at 8000 g for 10 min, the supernatant was collected, and 10 μl was assayed in a Luminoskan Type 391 (Labsystems, Finland). 50 μl assay substrate (Luciferase Assay System) was used for each reaction.

1. Bagel S, Hullen V, Wiedemann B, Heisig P (1999) *Impact of gyrA and parC mutations on quinolone resistance, doubling time, and supercoiling degree of Escherichia coli*. *Antimicrob Agents Chemother* 43:868-875.

2. Preisler A, Mraheil MA, Heisig P (2006) *Role of novel gyrA mutations in the suppression of the fluoroquinolone resistance genotype of vaccine strain Salmonella Typhimurium vacT (gyrA D87G)*. *The Journal of antimicrobial chemotherapy* 57:430-436.
